# Human RNA ligase 1 as a novel regulator of ribosome function and translation under oxidative stress

**DOI:** 10.1093/nar/gkag528

**Published:** 2026-06-08

**Authors:** Florian Michael Stumpf, Marissa Glauner, Jasmin Jansen, Virginie Marchand, Yuri Motorin, Florian Stengel, Andreas Marx

**Affiliations:** Department of Chemistry, University of Konstanz, Konstanz 78457, Germany; Konstanz Research School Chemical Biology, University of Konstanz, Konstanz 78457, Germany; Department of Chemistry, University of Konstanz, Konstanz 78457, Germany; Konstanz Research School Chemical Biology, University of Konstanz, Konstanz 78457, Germany; Konstanz Research School Chemical Biology, University of Konstanz, Konstanz 78457, Germany; Department of Biology, University of Konstanz, Konstanz 78457, Germany; Université de Lorraine, SMP IBSLor, EpiRNA-Seq core facility, 54000 Nancy, France; Université de Lorraine, SMP IBSLor, EpiRNA-Seq core facility, 54000 Nancy, France; Konstanz Research School Chemical Biology, University of Konstanz, Konstanz 78457, Germany; Department of Biology, University of Konstanz, Konstanz 78457, Germany; Department of Chemistry, University of Konstanz, Konstanz 78457, Germany; Konstanz Research School Chemical Biology, University of Konstanz, Konstanz 78457, Germany

## Abstract

Human RNA ligase 1 (Rlig1) is a recently identified human 5′–3′ RNA ligase required for maintaining 28S ribosomal RNA integrity and promoting cell survival under oxidative stress. Although its enzymatic activity suggests a role in RNA processing and repair, the broader molecular context of Rlig1 remains poorly defined. Here, we identified potential Rlig1-associated proteins by affinity enrichment–mass spectrometry. Subsequent analysis revealed proteins involved in RNA surveillance and processing, including RNA-binding and end-processing enzymes, and indicated strong enrichment of ribosomal proteins. We showed that Rlig1 interacts with 80S ribosomes *in vitro*. Consistent with this observation, polysome profiling revealed recruitment of Rlig1 to ribosomal fractions under oxidative stress. Functionally, Rlig1-knockout (KO) HEK293 cells exhibited accelerated polysome loss and significantly reduced global protein synthesis compared to wild-type (WT) HEK293 cells during oxidative stress. In addition, we showed that stress-induced RNA fragments containing a 5′-PO_4_ end accumulated in Rlig1-KO cells. Among these, transfer RNA halves were prominently enriched. Together, our study links Rlig1 to ribosomal complexes and suggests that Rlig1 contributes to preserving RNA integrity and supporting translational capacity during oxidative stress.

## Graphical Abstract

**Graphical Abstract:**
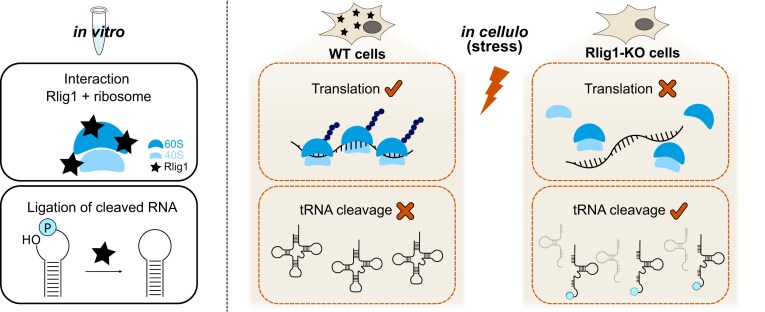

## Introduction

DNA repair has been extensively studied over the past decades, significantly advancing our understanding of genome maintenance. By now, a large number of DNA repair pathways have been described that guarantee the integrity of the genome [1–3]. In contrast, RNA repair remains poorly characterized. The prevailing view holds that damaged RNA is primarily eliminated through degradation, followed by resynthesis from an intact genomic template [4, 5]. The only two mechanisms of RNA repair known to date are the reversal of methylations, such as 1-methyl adenosine (m^1^A) and 3-methyl cytosine (m^3^C) by the AlkB family [6] and the removal of RNA–protein crosslinks [7–9]. However, not only DNA but also specific RNA types have been shown to exist and persist in cells for longer periods of time [10–12]. Several studies have shown that RNA is susceptible to certain types of cellular damage at an even higher rate than DNA [13–15]. Moreover, RNA is estimated to be ~4-fold more abundant than DNA in mammalian cells [16]. Thus, repairing damaged RNA, rather than degrading and resynthesizing it, could be energetically favourable for the cell, particularly in the case of long-lived RNAs [9]. The recent discovery of the 5′–3′ human RNA ligase Rlig1 supports the assumption that additional, yet unidentified RNA repair mechanisms could exist in human cells [17].

RNA ligase 1 (Rlig1) is the first described human 5′–3′ RNA ligase, able to fuse RNA strands with 5′-PO_4_ ends to 3′-OH ends [17]. As a member of the nucleotidyltransferase family, characterized by its conserved sequence motif KxxG in the active centre, the ligation mechanism of Rlig1 operates via a three-step mechanism [17–19]. Rlig1 is able to autoAMPylate itself at the position of lysine K57 located within its active site. Subsequently, Rlig1 transfers the AMP-moiety onto the 5′-PO_4_ of the first RNA strand that is to be ligated. This is followed by a subsequent nucleophilic attack by the 3′-OH of the second RNA strand on the formed RNA-adenylate resulting in the ligated RNA product.

We previously reported that Rlig1-deficient HEK293 cells exhibited increased degradation of 28S ribosomal RNA (rRNA) and reduced viability following menadione-induced oxidative stress, compared to WT cells [17]. Similar effects were observed upon chemical inhibition of Rlig1 [20]. Further research demonstrated that, in addition to menadione, a small number of other specific compounds were also capable of reducing the viability of HEK293 Rlig1-KO cells compared to WT cells [21]. Consequently, Rlig1-deficient HEK293 cells proved to be more susceptible to certain cellular stressors than HEK293 WT cells, supporting the hypothesis of a role for Rlig1 in RNA repair. Moreover, recent studies reported altered transfer RNA (tRNA), small nucleolar RNA (snoRNA), and small nuclear RNA (snRNA) levels in the brains of Rlig1-KO mice, [22] showed that Rlig1 is integral for circRNA biogenesis during viral infections [23] and that it promotes proliferation of triple-negative breast cancer cells by interacting with ERK, highlighting its potential oncogenic role [24].

Although Rlig1 has been implicated in RNA repair, the cellular role of the RNA ligase remains largely unclear. Here, we investigate the cellular function of Rlig1 in human cells using affinity enrichment–mass spectrometry, ribosome association, translation assays, and an RNA sequencing approach in WT and Rlig1-KO HEK293 cells. Our affinity enrichment analysis identified ribosomal proteins as prominent candidate interactors of Rlig1. Guided by these results, we found that Rlig1 associates with ribosomes i*n vitro* and *in cellulo*. Furthermore, Rlig1-deficient cells exhibited reduced translational activity and accumulated specific RNA fragments, including tRNA halves, under stress conditions. Our data suggest that Rlig1 promotes RNA integrity and efficient translation during oxidative stress [17, 25–27].

## Materials and methods

### General procedures

#### Cell culture

HEK293 WT and Rlig1-KO cells were cultured in Dulbecco’s modified Eagle’s medium GlutaMAX + 10% FCS in 15 cm standard cell culture plates (Sarstedt). The cells were incubated at 37°C, 7.5% CO_2_, and 95% humidity and passaged every other day.

#### SDS–PAGE

For the analysis of protein samples via sodium dodecyl sulphate–polyacrylamide gel electrophoresis (SDS–PAGE), 6× SDS loading dye was added to the samples to reach a final concentration of 1×. Subsequently, the samples were heated and denatured (95°C, 5 min). The proteins were separated utilizing a PAGE gel consisting of a stacking gel (5% acrylamide) and a resolving gel (12.5% acrylamide). Gel electrophoresis was performed with a Bio-Rad system in 1× SDS running buffer [25 mM Tris–HCl, 200 mM glycine, 0.1% (w/v) SDS] at 35 mA for one gel or 65 mA for two gels until the desired separation was achieved. As marker, a pre-stained protein ladder (Thermo Scientific) was used. The resulting gel with the separated proteins was subsequently stained with Krypton™ Fluorescence Protein Stain (Thermo Scientific) according to the manufacturer’s instructions or transferred on a polyvinylidene fluoride (PVDF) membrane (0.2 µm; GE Healthcare).

#### Western blot

The proteins were separated via SDS–PAGE as described above. Transfer of proteins from the gel onto a PVDF membrane (0.2 µm; GE Healthcare) was performed in 1× western blot transfer buffer (25 mM Tris–HCl, pH 8.3, 100 mM glycine) for 90 min at 60 V. After the transfer, the membrane was blocked with 5% milk in phosphate-buffered saline (PBS) with 0.1% Tween 20 (PBS-T) for 1 h. Then the membrane was incubated with the respective primary antibody (Rlig1 (sc-390730, Santa Cruz Biotechnology), RPL10L (ABC-AP17603a, Abcepta), RPS20 (ab133776, Abcam), or puromycin (MABE343, Merck) according to the manufacturer’s instructions. After washing the membrane (3 × 10 min, PBS-T), the suiting secondary antibody (1:20 000 in PBS-T, α-mouse, or α-rabbit) was added to the membrane and incubated for 1 h. Afterwards, the membrane was washed again (3 × 10 min, PBS-T) and incubated with Pierce™ ECL Western Blotting Substrate (Thermo Scientific) or SuperSignal™ West Femto Maximum Sensitivity Substrate (Thermo Scientific). Then, the membrane was subjected to chemiluminescence imaging with an Amersham imager 600 (GE Healthcare).

#### BCA determination of protein concentration

For the determination of protein concentration in a sample, the Pierce™ BCA (Bicinchoninic acid) Protein Assay Kit (Thermo Scientific) was utilized according to the manufacturer’s protocol. The calibration was achieved by diluting BSA in the same buffer as the samples in a range of 0.125–2.000 mg/ml. For the assay, 10 µl of each sample or standard was mixed with 200 µl of the provided BCA solution in a 96-well plate. The mixture was incubated at 37°C for 30 min in the dark and subsequently read out with a Victor^3^ 1420 Multilabel Counter (PerkinElmer).

### Elucidation of protein–protein interaction partners of Rlig1

#### Expression and purification of recombinant Rlig1 variants

The Rlig1 variants were expressed as previously reported [17].

#### Cell pellet production

For the cell pellets, 8 × 10^6^ cells were seeded in 20 ml medium in 15 cm cell culture plates. After 48 h at ~90% confluency, the cells were harvested. For stressed cells, the medium was removed and 10 ml new medium containing 40 µM menadione in EtOH (0.1%) was added and incubated (1 h, 37°C). Afterwards, the medium was removed and 5 ml ice-cold PBS was added to the cells. For unstressed cells, the incubation with menadione was omitted and 5 ml ice-cold DPBS was directly added after removing the medium. The cells were harvested with a cell scraper and centrifuged (10 min, 500 *g*, 4°C). The resulting pellets were resuspended in 400 µl ice-cold DPBS and the cells of four plates were combined and centrifuged (10 min, 500 *g*, 4°C). After removing the DPBS, the cell pellet was stored frozen (−80°C).

#### Lysate preparation for affinity enrichment

Directly before the experiment the frozen cell pellet was lysed by resuspending it in 750 µl ice-cold lysis buffer [25 mM Tris, pH 7.5, 150 mM NaCl, 1 mM ethylenediaminetetraacetic acid (EDTA), 1% (v/v) NP-40, 1 mM dithiothreitol (DTT), 0.1 M Pefabloc^®^ SC, 1 µg/ml aproptinin/leupeptin] and incubation on ice for 20 min. After the incubation, the lysate was cleared by centrifugation in an Eppendorf 5430R centrifuge (22 000 *g*, 30 min, 4°C). The protein concentration of the supernatant was determined by BCA as described above and adjusted to 6.67 mg/ml with lysis buffer. Subsequently, RNase If (6000 U/ml in lysis buffer) was added to the cell lysate (end concentration 600 U/ml with 6 mg/ml protein concentration) and incubated in a thermomixer (15 min, 37°C, 850 rpm) to result in the RNA digested cell lysate. The RNA digestion was controlled via a 4150 TapeStation system (Agilent).

#### Tapestation control of the RNA digested cell lysate

Two hundred fifty microlitres TRIzol™ reagent was given to 25 µl cell lysate in a 500 µl low-binding reaction tube. The mixture was vortexed (1 min) and incubated at RT (5 min). Afterwards, chloroform (250 µl) was added and the mixture was again vortexed (1 min) and incubated at RT (5 min). The sample was centrifuged (12 000 *g*, 15 min, 4°C) and the upper aqueous phase was transferred into a new 500 µl reaction tube. To this aqueous phase, chloroform (62.5 µl) was added and the mixture was vortexed (1 min) and incubated at RT (5 min) before being centrifuged again (12 000 g, 15 min, 4°C). The upper aqueous phase was transferred into a new 500 µl reaction tube and ice-cold isopropanol (170 µl) was added. The mixture was vortexed (1 min), flash frozen in liquid nitrogen, and incubated (−80°C, 18 h). The sample was carefully thawed and centrifuged (20 700 g, 30 min, 4°C), and the supernatant was discarded. To the transparent pellet ice-cold 80% EtOH (250 µl) was added and the sample was vortexed (1 min) and centrifuged again (20 700 g, 30 min, 4°C). After discarding the supernatant, the white RNA pellet was air dried (10 min) and dissolved in MQ water (40 µl). The resulting sample was analysed via an HSRNA ScreenTape, following the manufacturer’s protocol on a 4150 TapeStation system (Agilent).

#### Affinity enrichment using Rlig1 as bait

For the affinity enrichment of Rlig1, Strep-Tactin^®^ Superflow^®^ bead slurry (50 µl, IBA Lifesciences) was prepared in 500 µl low-binding reaction tubes. The beads were equilibrated (3 × 400 µl) with washing buffer (25 mM Tris, pH 7.5, 50 mM NaCl, 1 mM DTT) and kept on ice. His_6_-Strep-AMP-Rlig1 or His_6_-Strep-K57A-Rlig1 (100 µl, 0.5 mg/ml in washing buffer) was added to the equilibrated beads (50 µl). Washing buffer (100 µl) was used as negative control. The bead suspension was incubated in an overhead shaker (60 min, 4°C). Washing buffer (400 µl) was added to the beads and the sample was transferred to Mobicol spin columns (MoBiTec). The reaction tube that contained the beads was rinsed one time with washing buffer (100 µl) and the bead containing solutions were combined. The beads were washed (4 × 500 µl, washing buffer) and the RNA digested cell lysate (250 µl, 6 mg/ml) was added to the prepared beads and incubated on an overhead shaker (20 h, 4°C). The beads were washed (5 × 500 µl, washing buffer) and the protein was eluted with elution buffer (50 mM NH_4_HCO_3_, 0.8 mM biotin) (4 × 100 µl, 10 min, 37°C, 800 rpm). The eluate was dried in a SpeedVac (2 h, vacuum, no heating) and stored at −20°C.

#### Tryptic in-solution digest

The dried samples were dissolved in urea (100 µl, 8 M). Tris(2-carboxyethyl)phosphine (5 mM) was added and the samples were incubated (30 min, 37°C, 600 rpm). Afterwards, chloroacetamide (10 mM) was added to the solution and incubated (30 min, 23°C, 600 rpm, in the dark). The solution was diluted with NH_4_HCO_3_ (700 µl, 50 mM) and the pH was controlled to be between pH 7 and 8. Sequencing grade modified trypsin [2.5 µg, Promega (cat. nr. V5111)] was added, and the sample was incubated (20 h, 37°C, 600 rpm). Trifluoroacetic acid (TFA) in H_2_O [10% (v/v), 5 µl, LC–MS grade] was added and the sample was freeze-dried.

#### Desalting of the peptides

The freeze-dried peptides were dissolved (0.1% TFA in 5% acetonitrile (ACN), 40 µl) and 10% TFA (10 µl) was added. The pH was checked to be pH < 4. Pierce™ C18 Spin Tips (cat. nr. 84 850) were wetted (0.1% TFA in 80% ACN 20 µl) and equilibrated (0.1% TFA, 3 × 20 µl). The sample was loaded and the flow-through was loaded a second time for better retention. The tip was washed two times (0.1% TFA, 20 µl) and the bound peptides were eluted (0.1% FA in 80% ACN, 3 × 20 µl). The combined elution was dried in a SpeedVac (45 min, vacuum, no heat).

#### Affinity enrichment–mass spectrometry

For MS measurement the samples were dissolved (0.1% FA, 15 µl). The desalted peptide samples were analysed on an EASY-nLC 1200 UHPLC system (Thermo Scientific) coupled to a Q-Exactive HF mass spectrometer (Thermo Scientific) and separated on a C18 Acclaim PepMap RSLC column (50 µm × 15 cm, 2 µm, 100 Å, Thermo Scientific) with a flow rate of 300 nL/min over a 120 min gradient. After starting for 4 min with 94% buffer A (0.1% FA) and 6% buffer B (80% ACN + 0.1% FA), the concentration of buffer B was increased to 44% over 105 min, then set to 56% over 5 min, and finally increased to 100% buffer B over 1 min, followed by washing and re-equilibration of the column for 5 min. The samples were measured in data independent acquisition (DIA) mode using a total of 22 isolation windows of variable window size. The isolation width and window positions were adapted from [25]. MS1 scans were acquired between 300 and 1650 m/z with a resolution of 120 000 at 200 m/z. A maximum injection time of 60 ms and an AGC target of 3e6 was used. After one MS1 scan the mass spectrometer cycled once through all DIA windows. Isolated ions were fragmented via higher-energy collisional dissociation with stepped normalized collision energies of 25.5, 27, and 30 eV and fragment ion spectra were acquired with automatic injection time at a resolution of 30 000 at 200 m/z and an AGC target of 3e6. Spectra were recorded with a fixed first mass of 200 m/z.

#### Data analysis of affinity enrichment

The raw data from the MS measurements were first analysed with Spectronaut V16. Here, identification and label-free quantification of proteins was performed in library-free direct DIA mode. The default settings were used with a minimum peptide length of 5 AAs. ‘Uniprot_sprot_2022_01_07_HUMAN.fasta’ (downloaded 7 January 2022) and ‘Frankenfield2022_Universal_Contaminants.fasta’ were used as databases. The results were exported from Spectronaut and all further analyses of the raw label-free quantification (LFQ) data was performed with Perseus V1.6.15.0. Contaminants were filtered out using the contamination database and the LFQ intensities were log_2_(×) transformed. Only proteins that were consistently identified with a minimum of three precursor peptides in all three replicates of at least one condition were subjected to further evaluation. Missing values were imputed from a normal distribution (width = 0.3, shift = 1.8) and an ANOVA multiple sample test was conducted (S_0_ = 0.1, FDR = 0.01), followed by a Post hoc Tukey’s test (FDR = 0.01). Proteins enriched solely for the bead control according to the Post hoc test were filtered out before further processing. Intensities were normalized by z-scoring and the mean average of the triplicate measurements for each bait/condition was calculated. Only proteins with a minimum Z-score of >0.5 for the AMP-Rlig1 bait and a Z-score <0 for the bead control after the ANOVA analysis were considered as enriched for AMP-Rlig1 and considered for further analysis. GO-term analysis was performed with the DAVID tool [26]. The mass spectrometry proteomics data have been deposited to the ProteomeXchange Consortium via the PRIDE partner repository [27] with the dataset identifier PXD067856.

### Ribosomal experiments

#### Ribosome purification

For the purification of ribosomes, 5 ml HEK293 cell pellet was resuspended in 25 ml lysis buffer (50 mM HEPES, pH 7.4, 300 mM NaCl, 6 mM MgCl_2_, 0.5% NP40, 20 µM phenanthroline, 1 mM PMSF, 2 mM DTT, 1× cOmplete™ EDTA-free Protease inhibitor (Roche)) and incubated (30 min, 4°C, 400 rpm). After incubation with puromycin (1 mM, 30 min, 4°C), the lysate was cleared by two centrifugation steps (12 000 *g*, 10 min, 4°C). The cleared lysate was loaded on a 60% sucrose cushion [60% sucrose in 50 mM HEPES, pH 7.4, 50 mM KCl, 10 mM MgCl_2_, 5 mM EDTA, 1× cOmplete™ EDTA-free Protease inhibitor (Roche)]. For 8 ml of the cleared lysate, 12 ml sucrose cushion was used. Afterwards, the ribosomes were pelleted by centrifugation (184 000 *g*, 20 h, 4°C). The 80S ribosome pellet was resuspended (50 mM HEPES, pH 7.4, 300 mM KCl, 1 mM DTT) and the concentration for the 80S ribosomal fraction in µM was determined by multiplying the A260 absorbance measured via Nanodrop ND-1000 (Thermo Fisher) with the factor 0.02.

#### Ribosome sedimentation

80S ribosomes and Rlig1 were mixed in different concentrations in the reaction buffer [30 mM HEPES, pH 7.4, 100 mM KAc, 5 mM MgCl_2_] in a total volume of 35 µl and incubated (15 min, 400 rpm, 12°C). Afterwards, 30 µl of the sample was loaded onto a sucrose cushion (150 µl, 25% sucrose) in polycarbonate centrifuge tubes (Beckman Coulter, 7 × 20 mm) and centrifuged (90 min, 220 000 *g*, 4°C). After removing the sucrose, the resulting ribosomal pellet was carefully resuspended in 1× Laemmli sample buffer (30 µl, 30 min, 23°C, 600 rpm). Afterwards, more 1× Laemmli sample buffer was added (30 µl) and the sample was denatured (5 min, 95°C). For the samples before the sedimentation, the remaining 5 µl reaction solution were diluted with 2× Laemmli sample buffer (5 µl) and the sample was denatured (5 min, 95°C). The samples were analysed by PAGE and western blot.

#### Crosslinking experiment

Seventeen point five micromolare Rlig1-AMP or apo-Rlig1 K57A was gently mixed with 67 nM 80S ribosomes in crosslinking buffer (50 mM HEPES, pH 7.50, 5 mM MgCl_2_, 100 mM KCl). The mixture was incubated without agitation to allow complex formation (30 min, 37°C). Afterwards, 1.5 mM BS3-H12/D12 crosslinker (Creative Molecules Inc.) was added and the reaction mixture was incubated with gentle agitation (30 min, 37°C, 350 rpm). After the crosslinking, unreacted linker was quenched by the addition of NH_4_HCO_3_ (50 mM, 15 min). The samples were dried and subsequently dissolved in 8 M urea. The sample was reduced with Tris(2-carboxyethyl)phosphine (2.5 mM, 30 min, 37°C), alkylated with iodoacetamide (5 mM, 30 min, RT, in the dark), and digested with trypsin (Promega, 1:50 enzyme-to-substrate, 16 h, 37°C). The peptides were desalted [C18 Sep-Pak cartridges (Waters)] and crosslinked peptides were enriched by size exclusion chromatography using an ÄKTA micro chromatography system (GE Healthcare) equipped with a SuperdexTM Peptide 3.2/30 column. Fractions containing peptides were collected and combined to four fractions. LC–MS^2^ analysis was performed on an Orbitrap Tribrid Fusion mass spectrometer coupled to an EASY-nLC 1200 system (Thermo Scientific). Peptides were separated across a 75-min gradient on an Acclaim PepMap column (150 × 0.05 mm, 2 µm, Thermo Scientific, P/N 164943). MS measurement was performed in data-dependent mode with a maximum cycle time of 3 s. Full scans were acquired in the Orbitrap at a resolution of 120 000, in the scan range of 400–1500 m/z, AGC target 2e5 and a maximum injection time of 50 ms. For precursor selection, monoisotopic precursor selection was set to peptides, dynamic exclusion to 60 s and only precursor ions with charge states 3–8 were isolated for MS/MS acquisition. Precursor ions were fragmented with 35% CID activation and MS/MS scan was acquired in the iontrap in rapid scan mode. Crosslink analysis was done with xQuest/xProphet [28] in ion-tag mode with a precursor mass tolerance of 10 ppm. For matching of fragment ions, tolerances of 0.2 Da for common ions and 0.3 Da for crosslinked ions were applied. The database was compiled of the Rlig1 protein sequence as well as proteins that are part of the mature cytosolic ribosome. An FDR was estimated by xProphet and only hits with an FDR below 0.01 were used. The experiment was performed as biological triplicate with two technical replicates each. Crosslinks identified with ld-score ≥25 and deltaS <0.95 were retained for further analysis and interlinks were filtered with the mi-filter [29]. The crosslinking data was visualized by xiNET [30]. The XL-MS data have been deposited to the ProteomeXchange Consortium via the PRIDE partner repository [27] with the dataset identifier PXD067615.

### Polysome profiling

#### Preparation of the sucrose gradient

Sucrose solution [5% and 50% (w/v)] in polysome profiling buffer [20 mM Tris, pH 7.4, 100 mM KCl, 10 mM MgCl_2_; complete EDTA-free protease inhibitor cocktail (Roche), 100 µg/ml cycloheximide, 2 mm DTT, 50 U/ml RNasin (Promega)] was prepared. A linear gradient was generated in ultracentrifuge tubes (14 × 89 mm, PC, Seton Scientific) using a Gradient Master device (BioComp) running the ‘SW41 Short Gradient’ program.

### Polysome isolation and fractionation

Two days prior the experiment, 6 × 10^6^ HEK293 WT or Rlig1-KO cells were seeded in 15 cm cell culture dishes as described in the ‘Cell culture’ section. At 80% confluency, the cells were treated with menadione (40 or 60 µM) for oxidative stress induction for 1 h. As control, no menadione was added to an additional cell dish. Subsequently, the cells were incubated with cycloheximide (100 µg/ml, 5 min). Afterwards, the medium was removed and 10 ml ice-cold phosphate-buffered saline supplemented with cycloheximide (100 µg/ml) was added. Cells were harvested by scraping on ice and collected by centrifugation (500 *g*, 10 min, 4°C). Cell pellets were stored at −80°C until further processing.

For the polysome isolation, the cells were resuspended in lysis buffer [5 mm Tris–HCl, pH 7.5, 2.5 mm MgCl_2_, 1.5 mm KCl, complete EDTA-free protease inhibitor cocktail (Roche), 100 µg/ml cycloheximide, 2 mm DTT, 50 U/ml RNasin (Promega)] and incubated on ice for 20 min. The cell suspension was vortexed every 5 min. Subsequently, the lysate was cleared by centrifugation (16 000 *g*, 5 min, 4°C). For each sample, the absorption at 260 nm was determined and the lysate concentration was adjusted to A_260_ = 20 with lysis buffer for each sample. Five hundred microlitres of the lysate was loaded on the pre-prepared sucrose gradients and ultracentrifuged (36 000 rpm, 2 h, 4°C) using a SW41Ti rotor (Optima L-90K Ultracentrifuge, Beckman Coulter).

For the recording of the polysome profiles, a Piston Gradient Fractionator (Biocomp) was used. The profiles were recorded by measuring the absorption at 260 nm from the top to the bottom of the gradient. Fractions were collected automatically in a volume of 800 µl. Subsequently, 600 µl of the fraction was mixed with 600 µl ice-cold ethanol, snap-frozen in liquid nitrogen and stored at −80°C.

### Protein preparation for western blot analysis

The samples containing 50% EtOH were centrifuged (20 000 *g*, 30 min, 4°C), the supernatant was discarded and the protein pellet was washed with 80% EtOH (500 µl). The pellet was dried at 95°C for 3 min and the proteins were resuspended in 1× Laemmli sample buffer (fraction 1–2:250 µl; fraction 3–12:25 µl). The samples were denatured (95°C, 5 min) and analysed by SDS–PAGE and western blot as described above.

### Puromycin incorporation assay

One day prior to the experiment, 1.2 × 10⁶ HEK293 WT or Rlig1-KO cells were seeded in 6-well plates and incubated as described in the ‘Cell culture’ section. For oxidative stress induction, cells were treated with 40 µM menadione for 1 h. For the final 15 min of treatment, 5 µM puromycin was added to the well to label newly synthesized polypeptides. Control cells were incubated with puromycin alone. Following the treatment, cells were harvested, and puromycin incorporation was visualized by SDS–PAGE and immunoblotting as described above. Quantification of protein synthesis rate was achieved by calculating the ratio of puromycin signal intensity to total protein levels that were determined by a separate Coomassie staining. Protein synthesis levels in untreated HEK293 WT cells were defined as 100%, and all other conditions were normalized relative to this reference.

### 5′-PO_4_ sequencing

#### Total RNA extraction

One day prior to the experiment, 1.2 × 10⁶ HEK293 WT or Rlig1-KO cells were seeded in 6-well plates and incubated as described in the ‘Cell culture’ section. For oxidative stress induction, cells were treated with 40 µM menadione for 3 h and harvested. Cell pellets were lysed in TRIzol reagent (1 ml per pellet; Invitrogen) and incubated for 5 min at RT. Chloroform (250 µl per 1 ml TRIzol) was added, samples were vortexed (10 min), and phase separation was achieved by centrifugation (12 000 g, 15 min, 4°C). The aqueous phase was collected, re-extracted with chloroform, and RNA was precipitated by adding cold isopropanol (1:2, v/v) followed by incubation at −80°C overnight. RNA was pelleted (20 000 *g*, 30 min, 4°C), washed twice with 80% ethanol, air-dried, and dissolved in MQ water.

#### Preparation of complementary DNA library for 5′-PO_4_ sequencing

Total RNA (200 ng) was incubated in bicarbonate buffer (100 mM bicarbonate, pH 9.2, 5 min, 96°C). The reaction was stopped by addition of EtOH (1 ml), 3 M sodium acetate (pH 5.2; 10 µl), glycoblue (1 µl). After snap freezing in liquid nitrogen, the samples were centrifuged (full speed, 30 min, 4°C). RNA pellets were washed with EtOH (80%) and again centrifuged (full speed, 10 min, 4°C). RNA was resuspended in MQ water (24 µl). For 3′ dephosphorylation, RNA was mixed with dephosphorylation mixture [25 µl; 200 mM Tris–HCl, pH 6.5, 200 mM magnesium acetate, 10 mM β-mercaptoethanol, 1 µl T4 PNK (NEB)] and incubated (37°C, 6 h). For inactivation, the mixture was heated to 65°C for 20 min. Afterwards, RNA fragments were purified (RNeasy MinElute Cleanup kit (Qiagen)) and eluted in MQ water (10 µl). RNA was converted to library using NEBNext^®^ Small RNA Library kit (NEB) following the manufacturer’s instructions. Amplified libraries were purified (GeneJet PCR kit, Thermo Scientific), assessed on a TapeStation D1000 chip, quantified by Qubit fluorometry, multiplexed, and sequenced on an Illumina NextSeq2000 (50 bp single-end mode).

#### Data analysis

High-quality raw sequencing reads (>Q30) were subjected to trimming using Trimmomatic v0.39 with the following parameters: MINLEN:08, STRINGENCY:7, AVGQUAL:30 [31]. Trimmed reads were aligned to the *Homo sapiens* combined rRNA/tRNA reference sequence using bowtie2 v2.4.4 in end-to-end mode (--no-unal --no-1mm-upfront -D 15 -R 2 -N 0 -L 10 -i S, 1, 1.15 as other bowtie2 parameters), only uniquely mapped reads in positive orientation were retained for further analysis [32]. Reads’ extremities 5′-ends were counted for each RNA position in the reference, this reads’ count was used as the measure for intensity of cleavage at a given position. Three scores were used for analysis of 5′-P Seq raw data (derived from AlkAniline Seq scores): Normalized cleavage (NCleavage), normalized count (NormCount), and stop ratio [33, 34]. Normalized cleavage corresponds to 1000× proportion of reads starting at a given position to the total number of reads mapped to a given RNA sequence. Stop ratio, closely derived from ψ-ratio used for analysis of Ψ-seq data, is calculated as a ratio of reads starting at a given position to total number of reads passing (covering) it [35]. In addition, NormCount uses local normalization to the median of cleavage signals in ±5 nt window around of analysed position. NormCount uses all cleavage signals in the window. This score represents the intensity of the cleavage at a given nucleotide compared to the local cleavage background in the adjacent RNA region. The raw sequencing data generated in this study have been deposited in the European Nucleotide Archive under project accession number PRJEB110276.

## Results

### Work-flow for the identification of putative Rlig1 interaction partners

**Figure 1.**
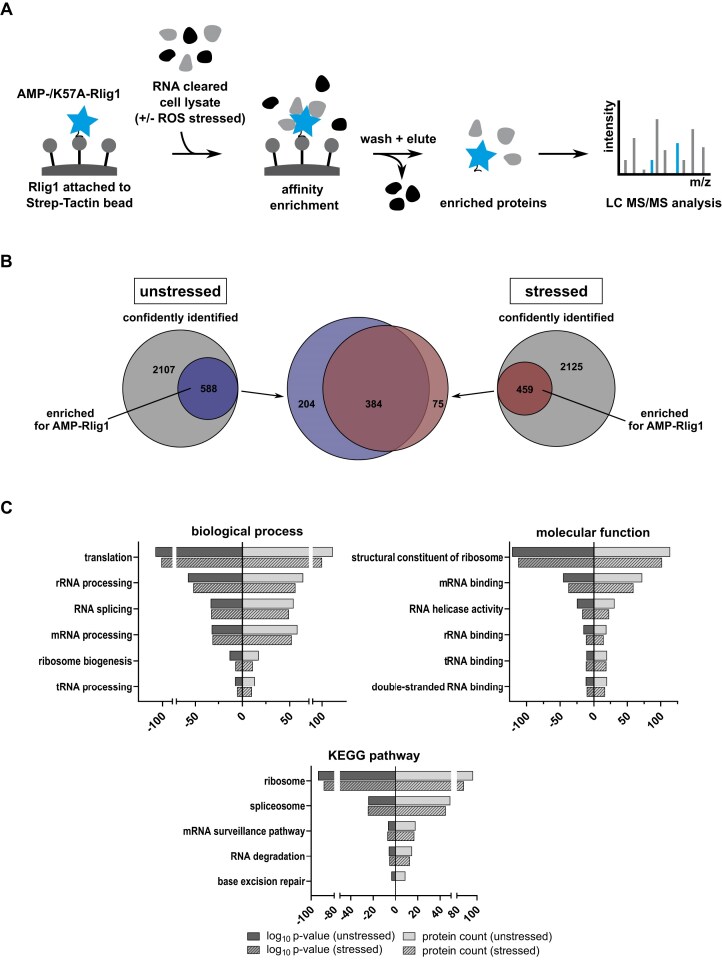
AMP-Rlig1-associated proteins are mainly involved in RNA-based processes and are highly similar for stressed and unstressed cells. (**A**) Work-flow for the identification of putative Rlig1 interaction partners by affinity enrichment–mass spectroscopy. (**B**) Venn diagram of identified and significantly enriched proteins for the active AMP-Rlig1 variant from HEK293 Rlig1-KO cells grown under physiological conditions (blue) or after treatment with menadione (red). Significant enrichment determined from biological triplicates by ANOVA multiple sample test (S_0_ = 0.1, FDR = 0.01) and Post hoc Tukey’s test (FDR = 0.01). (**C**) Classification of potential Rlig1 interaction partners according to GO term analysis. Shown are enriched biological processes, molecular functions and KEGG pathways based on DAVID classification [26]. Plotted are the log_10_ of the modified Fisher Exact *P*-value for the corresponding GO term and the count of proteins belonging to this GO term. Results for proteins enriched in unstressed and stressed samples were plotted separately as indicated by the legend.

To identify potential cellular interaction partners of Rlig1, we performed an affinity enrichment–mass spectrometry experiment using recombinant Rlig1 variants and lysates from HEK293 Rlig1-KO cells (Fig. 1A). This experiment was intended as an exploratory approach to identify candidate pathways and complexes that may be linked to Rlig1 function. We employed recombinantly expressed AMP-Rlig1 as the active bait and included the catalytically inactive K57A-Rlig1 variant, which cannot undergo spontaneous AMPylation during affinity enrichment, to distinguish activity-dependent from activity-independent associated proteins [17]. In addition, an uncharged bead control was included to differentiate between non-specific binding to the beads and specific interactions of Rlig1. Cell lysates from HEK293 Rlig1-KO cells were used to minimize competition between endogenous Rlig1 and the employed bait. Consistent with the previous experiments, lysates from unstressed and menadione-treated cells were used to examine potential stress-dependent changes in Rlig1-associated proteins [17]. Furthermore, the cell lysates were previously cleared from RNA to exclude indirect RNA-mediated interactors. Here, RNA digestion was optimized with the RNA endonuclease RNase I_f_, as it is a common and well-characterized RNA endonuclease that cleaves RNA dinucleotide bonds without sequence specificity [36]. The RNase concentration was optimized for a short incubation time (Supplementary Figs S1A and B, and S2A). Subsequently, the Rlig1 variants were immobilized on streptavidin conjugated agarose beads via a co-expressed Strep-tag and the loaded beads were incubated with the cell lysates that were previously cleared from RNA. After thorough washing of the beads, the enriched proteins were eluted with biotin and prepared for subsequent analysis by SDS–PAGE and MS-based proteomics.

### Identification of potential interaction partners of Rlig1

Eluted proteins were first analysed by SDS–PAGE and visualized by Krypton staining. Here, Rlig1-containing samples showed multiple additional protein bands in comparison to the empty bead control, clearly indicating a successful enrichment (Supplementary Fig. S2B and C). Furthermore, differences in the intensities of various bands in the enriched proteins of the stressed and unstressed approach could be detected in the gel. Eluted proteins were then subjected to a tryptic in-solution digest and analysed by MS based proteomics using label-free DIA for identification and quantification of potential Rlig1 interactors. Initial principal component analysis of all samples was able to effectively distinguish the bead control from the bait-containing samples in the first principal component (Supplementary Fig. S3). In addition, active AMP-Rlig1 samples were clearly separated from inactive K57A-Rlig1 samples. The stressed samples were effectively separated from the unstressed samples in the second principal component. The variance of the biological replicates proved to be significantly smaller than between the different conditions. Two thousand one hundred seven proteins were confidently identified for the unstressed and 2125 for the stressed samples (*q*-value <0.01 and identified in all three biological replicates with ≥3 peptides in at least one of the conditions; for details see the ‘Materials and methods’ section). From those, 588 proteins were significantly enriched by AMP-Rlig1 in the unstressed and 459 in the stressed samples (ANOVA FDR = 0.01, S_0_ = 0.1; Post hoc Tukey’s test FDR = 0.01) (Fig. 1B) (for details, see the experimental section, for the resulting protein lists see SI_List of significantly enriched proteins). The majority of proteins was enriched for both variants, albeit to a lesser extent for the inactive K57A-Rlig1 in most cases (Supplementary Fig. S4). A large overlap of 384 proteins existed between the stressed and the unstressed samples (84% and 65% of the enriched proteins, respectively). Only 75 proteins were enriched exclusively in the stressed samples, while 204 proteins were solely enriched in the unstressed samples for AMP-Rlig1. Subsequent analysis of the AMP-Rlig1 enriched proteins revealed very similar significant GO terms for the proteins enriched in the two treatments (Fig. 1C), as well as for the proteins enriched exclusively in the stressed or unstressed samples (for the lists of GO terms for the different treatments see SI_List of enriched GO terms). Overall, our data therefore show a consistent set of cellular candidate interaction partners for Rlig1 after menadione treatment.

Proteins strongly enriched by AMP-Rlig1 under both stressed and unstressed conditions were predominantly associated with the biological processes of translation, RNA splicing, and the processing of diverse RNA species. With respect to molecular function, proteins binding various types of RNA as well as proteins exhibiting RNA helicase activity were notably enriched. Notably, most of the identified RNA helicases showed stronger enrichment with the active AMP-Rlig1 variant than with the inactive K57A-Rlig1 variant (Supplementary Fig. S5A). Among the significantly enriched KEGG pathways, the ‘messenger RNA (mRNA) surveillance pathway’ and ‘RNA degradation’ were prominent under both conditions, while ‘base excision repair’ was specifically enriched in unstressed cells. These findings are consistent with a potential role of Rlig1 in the RNA damage response. The majority of potential Rlig1 interactors assigned to the ‘RNA degradation’ category were components of the exosome. Notably, all but one of the known exosome subunits were enriched under both stressed and unstressed conditions (Supplementary Fig. S5B). Remarkably, DIS3, the catalytically active subunit of the exosome complex, [37] exhibited a complete shift in binding preference upon stress treatment. In unstressed samples, DIS3 was strongly enriched with the enzymatically active AMP-Rlig1 bait. In contrast, under oxidative stress, DIS3 was almost exclusively enriched with the inactive K57A-Rlig1 variant (Supplementary Fig. S5B). This shift in enrichment from the active AMP-Rlig1 variant under unstressed conditions to the inactive K57A-Rlig1 variant during oxidative stress was a rare observation in our dataset and, among the proteins examined in detail, represented the only instance of such a pronounced change in binding preference.

Within the ‘base excision repair’ category, several components of both the short-patch and long-patch base excision DNA repair pathways were enriched by Rlig1. These included, for example, the glycosylase MPG and the endonuclease APE1, both of which are known to process RNA in addition to their canonical roles in DNA repair [38–41].

Interestingly, the end-processing enzymes, ANGEL2 and CLP1 were significantly enriched with the active AMP-Rlig1 variant under both stressed and unstressed conditions (Supplementary Fig. S5C).

Furthermore, a significant enrichment of ribosomal proteins was observed in both the affinity enrichment dataset and the GO term analysis. Most of these ribosomal proteins displayed stronger enrichment with the active AMP-Rlig1 variant than with the inactive K57A-Rlig1 variant in both unstressed and stressed cells (Supplementary Fig. S5D). These observations prompted us to further investigate a potential association of Rlig1 with ribosomal complexes.

### Rlig1 interacts with the ribosome *in vitro*

**Figure 2.**
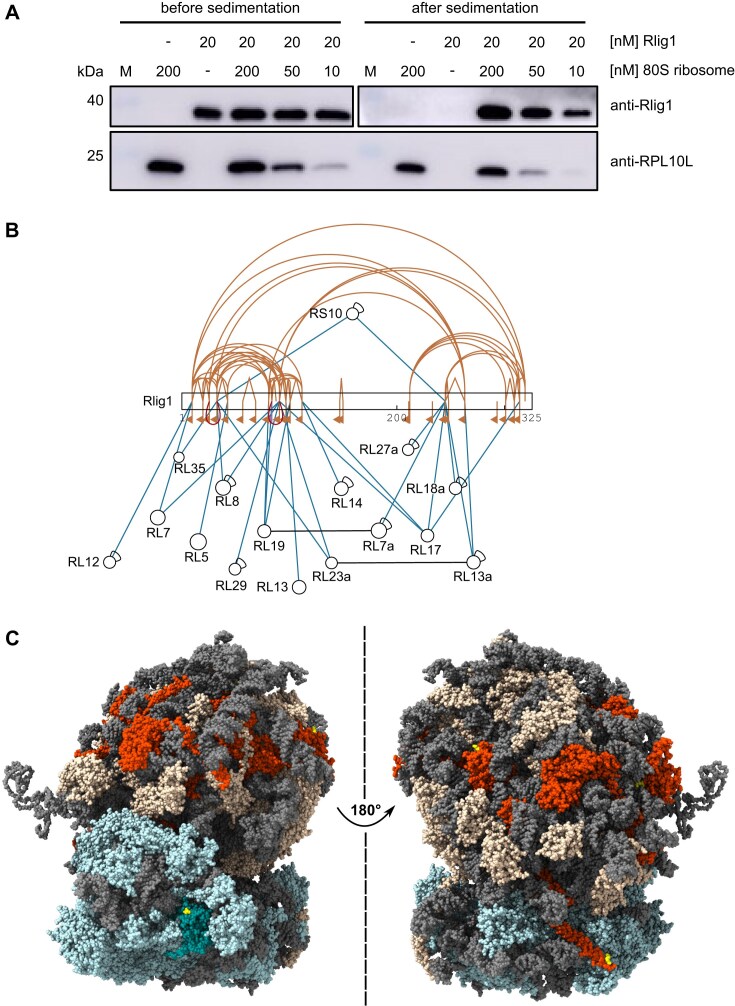
AMP-Rlig1 binds preferentially to the large ribosomal subunit of the 80S ribosome *in vitro*. (**A**) Co-sedimentation of AMP-Rlig1 with different concentrations of human 80S ribosome analysed by western blot. Anti-Rlig1 (top) and anti-RPL10L were used as control for the sedimentation of the 80S ribosome (bottom). Of note: the Rlig1 western blot after sedimentation was exposed for a longer time compared to the one before the sedimentation in order to result in a sufficient signal. (**B**) Crosslinking of AMP-Rlig1 with the 80S ribosome using the lysine reactive crosslinker BS3. Shown are only crosslinks between Rlig1 and ribosomal subunits that were confidently identified in 2 of 3 biologically independent replicates [29]. Rlig1 is shown in an opened sequence view while ribosomal proteins are indicated by black circles with arches indicating identified intra-protein crosslinks for this protein. Inter-protein crosslinks of ribosomal proteins to AMP-Rlig1 are shown in blue and intra-protein crosslinks of AMP-Rlig1 in orange. Linker modified lysines are shown as orange flags. Inter-protein crosslinks between ribosomal proteins are shown in black. (**C**) Cryo-EM map of the human 80S ribosome (PDB: 4UG0) [42] showing proteins and interaction sites crosslinked to Rlig1. 60S ribosomal proteins for which a crosslink to Rlig1 was identified are shown in dark orange and the crosslinked small subunit RS10 in teal. Lysines that were directly crosslinked to AMP-Rlig1 are depicted in yellow. 60S ribosomal proteins that were not crosslinked to AMP-Rlig1 are shown in beige, 40S ribosomal proteins that were not crosslinked to AMP-Rlig1 in light blue. The ribosomal RNA is depicted in grey.

To investigate a potential association of Rlig1 with the ribosome *in vitro*, we performed a ribosome sedimentation assay in which increasing concentrations of purified, intact human 80S ribosomes were incubated with recombinantly expressed AMP-Rlig1 and subsequently sedimented through a sucrose cushion. The intact 80S ribosome possesses sufficient mass to traverse the sucrose cushion, whereas AMP-Rlig1 alone is unable to sediment under these conditions. When AMP-Rlig1 was incubated with the ribosome prior to sedimentation, AMP-Rlig1 was detected in the pellet fraction (Fig. 2A). Moreover, the amount of detected AMP-Rlig1 correlated with the amount of 80S ribosome used in the assay. However, the RNA ligase did not sediment in an equimolar ratio with the ribosome, and the signal for Rlig1 in the sedimented samples was markedly reduced compared with the signal prior to sedimentation. Consequently, immunoblots showing the Rlig1 signal before and after sedimentation were imaged separately to prevent the stronger pre-sedimentation signals from obscuring the weaker post-sedimentation signals. Taken together, the results of this assay indicate an interaction of AMP-Rlig1 with the ribosome.

### Rlig1 interacts with the large ribosomal subunit *in vitro*

After confirming an association of Rlig1 with the ribosome, we next examined whether Rlig1 interacts with a specific region of the ribosome. To address this, an *in vitro* crosslinking experiment was performed using purified human 80S ribosome and recombinantly expressed AMP-Rlig1. The intact ribosome was preincubated with AMP-Rlig1 and subsequently crosslinked using the homobifunctional, amine-reactive crosslinker BS3 (bissulfosuccinimidyl suberate). After tryptic digestion, the resulting crosslinked peptides were analysed by MS/MS to identify specific interaction sites between the ribosome and the RNA ligase.

The identified interprotein crosslinks between Rlig1 and ribosomal proteins showed a pronounced preference for proteins of the large ribosomal subunit. Of the 16 crosslinked ribosomal proteins, only one, RS10, belongs to the small ribosomal subunit (Fig. 2B). However, examination of the remaining 15 proteins from the large ribosomal subunit that contained crosslinks did not reveal a discrete interaction site for AMP-Rlig1 on the subunit. The most apparent common feature was that the crosslinked ribosomal proteins are located in close proximity to rRNA on the ribosomal surface (Fig. 2C), as revealed by mapping the crosslinks onto the cryo-electron microscopy structure of the ribosome (PDB: 4UG0) [42].

### Rlig1 protects translational activity in HEK293 cells under menadione-induced stress

**Figure 3.**
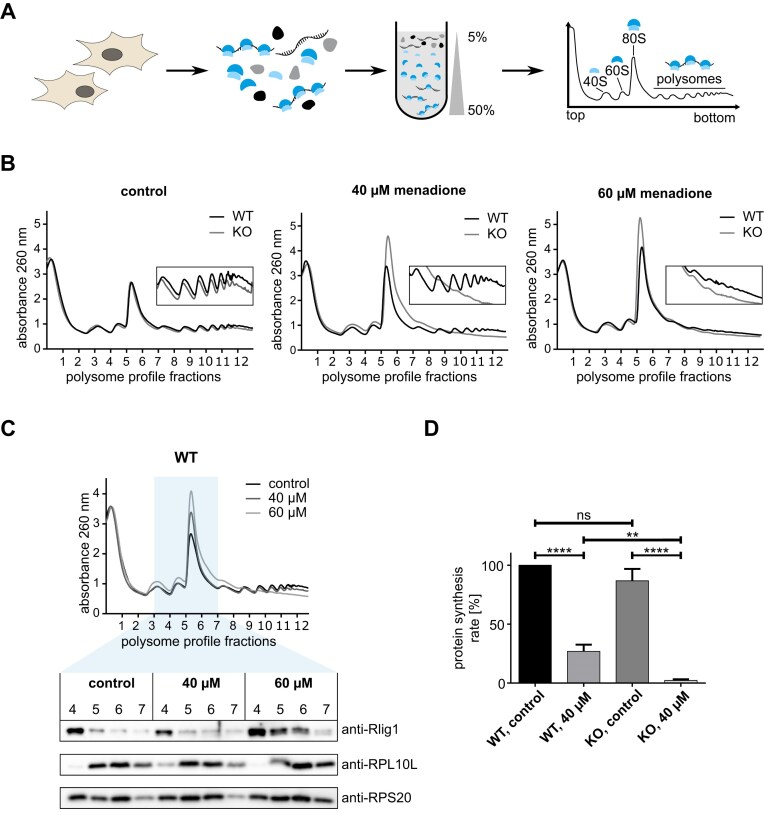
Rlig1 is important for maintenance of translational activity in cells under oxidative stress induced by menadione. (**A**) Schematic depiction of the polysome profiling work-flow. (**B**) Polysome profile of HEK293 WT (black) and Rlig1-KO cells (grey). Cells were grown under physiological conditions (control) or subjected to 40 or 60 µM menadione for 60 min at 37°C. Translation was arrested with cycloheximide. Cleared cell lysate was separated in a 5%–50% sucrose gradient by ultracentrifugation and analysed at 260 nm. (**C**) Combined polysome profiles of the WT cells treated with different concentrations of menadione (top). Corresponding western blot analysis of fractions 4–7 of the polysome profiles (bottom). Fractions analysed by western blot highlighted in blue. Antibodies were used against RPL10L as control for the 60S ribosomal subunit, RPS20 as control for the 40S ribosomal subunit and against Rlig1. Two independent experiments were performed with similar results. (**D**) Analysis of protein synthesis rate determined by puromycin incorporation assay in HEK293 WT or Rlig1-KO cells under physiological condition or after treatment with 40 µM menadione for 60 min. Protein synthesis rate was calculated by the ratio of puromycin signal intensity to total protein levels as determined by Coomassie staining. Synthesis rate of untreated HEK293 WT cells was set to 100% and all other values were calculated accordingly. Samples were measured in biological triplicates. Error bars represent the ±SD. Statistical significance was determined by two-way ANOVA. Significance levels are indicated as, **P < .01, ^****^P < .0001.

After further substantiating the binding of Rlig1 to the ribosome *in vitro*, we next investigated the potential functional consequences of this interaction and the effect of Rlig1 on ribosomal activity *in cellulo*. To this end, we analysed the translational activity of HEK293 WT and Rlig1-KO cells by polysome profiling under physiological conditions and following treatment with the ROS inducer menadione (Fig. 3A). Under physiological conditions, both cell lines displayed comparable polysome profiles characterized by multiple prominent polysome peaks (Fig. 3B). Remarkably, treatment with 40 µM menadione resulted in a pronounced loss of polysomes in Rlig1-KO cells, accompanied by a shift towards the peak corresponding to the 80S monosome and the peaks of the individual ribosomal subunits. This shift indicates reduced translation initiation and overall translational activity. In contrast, this effect was less pronounced in HEK293 WT cells, where polysome integrity was largely preserved (Fig. 3B and Supplementary Fig. S6A). Treatment with 60 µM menadione caused a loss of polysomes and accumulation of 80S ribosomes and individual ribosomal subunits in both cell lines (Supplementary Fig. S6A). However, the 80S peak remained more prominent in Rlig1-KO cells than in WT cells (Fig. 3B).

Subsequent immunoblot analysis of fractions obtained from polysome profiling of HEK293 WT cells was performed to determine the distribution of Rlig1 across the gradient. Under all conditions, the strongest Rlig1 signal was detected in the soluble, non-ribosomal fractions 1–3 (Supplementary Fig. S6B). Because of the high signal intensity in these fractions, the remaining fractions were analysed separately. To enable a direct quantitative comparison of Rlig1 levels between treatment conditions, fractions 4–7 from each condition were analysed on the same gel (Fig. 3C). These fractions correspond to the 40S ribosomal subunit, 60S ribosomal subunit, the 80S ribosome, and the initial polysome peak. However, due to automated fraction collection, individual fractions may span multiple adjacent peaks of the polysome profile, which prevents an exact assignment to specific ribosomal species. Under physiological conditions and following treatment with 40 µM menadione, immunoblot analysis indicated the presence of Rlig1 predominantly in fraction 4, which mainly represents the 40S ribosomal subunit. In contrast, after treatment with 60 µM menadione, Rlig1 was additionally detected in fractions 5 and 6, suggesting recruitment of Rlig1 to the 60S ribosomal subunit and the 80S monosome. This redistribution coincided with the observed loss of polysomes in HEK293 WT cells under these stress conditions.

To further elucidate the role of Rlig1 in cellular translation, global protein synthesis was assessed in HEK293 WT and Rlig1-KO cells under physiological conditions and under oxidative stress induced by 40 µM menadione. Translational activity was measured using puromycin labelling (Supplementary Fig. S6C). Under physiological conditions, no significant difference in global protein synthesis was observed between HEK293 WT and Rlig1-KO cells (Fig. 3D). Upon oxidative stress, however, both cell lines exhibited a marked reduction in protein synthesis. Notably, this decrease was significantly more pronounced in the Rlig1-KO cells compared to the WT cells. Together with the observed accumulation of 80S monosomes in Rlig1-KO cells under oxidative stress and the stress-dependent redistribution of Rlig1 to ribosome-associated fractions, these findings suggest that Rlig1 contributes to the maintenance of translational activity under menadione-induced stress conditions.

### Rlig1 deficiency results in rRNA and tRNA fragment accumulation under menadione-induced stress

To explore the molecular basis of this function, we next asked whether Rlig1 safeguards the integrity of RNAs required for translation. Building on the findings by Yuan *et al*. demonstrating reduced integrity of 28S rRNA in Rlig1-KO cells under menadione-induced stress, and our observation of decreased translation under similar conditions, we investigated whether the absence of Rlig1 leads to RNA strand breaks that require ligation activity under stress [17]. For Rlig1-mediated ligation, RNA breaks must carry a 5′-PO_4_ end and a 3′-OH terminus. Accordingly, we hypothesized that loss of Rlig1 results in the accumulation of RNA fragments bearing a 5′-PO_4_ that represent potential Rlig1 substrates.

**Figure 4.**
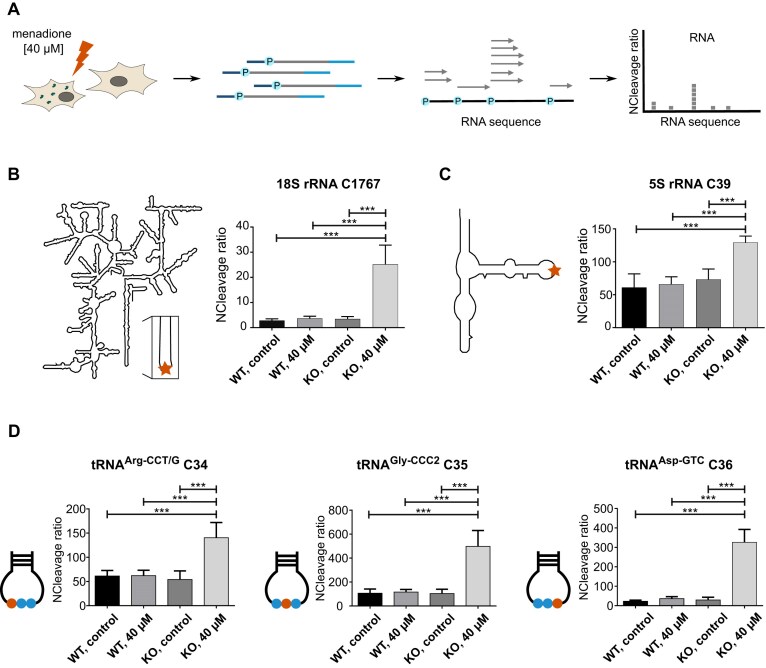
RNA fragments containing a 5′-PO_4_ end accumulate in Rlig1 deficient cells under stress. (**A**) Schematic depiction of 5′-PO_4_ sequencing work-flow and data analysis. RNA was extracted from HEK293 WT and Rlig1-KO cells under control (WT, control; KO, control) and menadione-treated conditions (WT, 40 µM; KO, 40 µM) (*n* = 5). Complementary DNA libraries were generated and sequenced. Reads were aligned to reference RNA sequences, and 5′-PO_4_ sequencing signal intensity was calculated as NCleavage ratio. Accumulated fragments and cleavage sites were identified by analysing the log_2_FC in NCleavage ratios of the KO stressed condition (KO, 40 µM) to the control group (WT control, WT 40 µM, KO control) using stringent filtering criteria (*P* < .05 and log_2_-fold change >0.5). (**B**) NCleavage ratios of 18S rRNA at position C1767 are shown for WT and Rlig1-KO cells under physiological conditions (control) or after stress treatment with 40 µM menadione for 3 h (40 µM) (right). Corresponding schematic secondary structure of 18S rRNA with the cleavage site highlighted (star, left). (**C**) NCleavage ratios of 5S rRNA at position C39 are shown (right). Corresponding schematic secondary structure of 5S rRNA with the cleavage site highlighted (star, left). (**D**) Representative examples of cleavage sites in the tRNA anticodon loop. NCleavage ratios for tRNA^Arg-CCT/G^ at position C34 (left), tRNA^Gly-CCC2^ at position C35 (middle), and tRNA tRNA^Asp-GTC^ at position C36 (right) are shown. Data represent five biological replicates. Error bars represent the ±SD. Statistical significance was assessed by two-way ANOVA (***P < .001).

To test this hypothesis, we employed an NGS-based approach designed to capture RNA fragments with a 5′-PO_4_ end (Fig. 4A). Total RNA was isolated from HEK293 WT and Rlig1-KO cells under control conditions and after treatment with 40 µM menadione for 3 h. Five biological replicates were prepared for each condition. Enrichment of 5′-PO_4_-containing RNAs was achieved through selective ligation of a 5′-adapter, which occurs only on RNA fragments carrying a natural 5′-PO_4_. Sequencing reads were aligned to reference RNA sequences, and normalized cleavage ratios (NCleavage ratio) were calculated from the number of reads sharing identical 5′-termini [(reads starting at a given position × 1000) / (total reads aligned to that RNA)] as described by Marchand *et al*. [43]. This analysis quantifies fragment accumulation and thereby identifies both the position and the relative intensity of cleavage events.

Cleavage sites were first examined under control conditions by calculating the log₂ fold change (log_2_FC) of the NCleavage ratio between WT and KO at each nucleotide position and filtering for significant sites (*t*-test, *P* < .05; log_2_FC > 0.5/1; NCleavage ratio >25). In control conditions, rRNA cleavage profiles revealed no significant difference in 5′-PO_4_ fragment accumulation between WT and Rlig1-KO cells. In contrast, a subset of tRNA species showed significantly elevated NCleavage ratios in the Rlig1-KO cells, with cleavage sites predominantly located in the T-loop region (Supplementary Fig. S7A). The strongest increases were detected for tRNA^Gly-CCC2^ and tRNA^Asp-GTC^. In both cases, cleavage occurred directly upstream of the T-loop-specific ψ-C motif (Supplementary Fig. S7B).

We then applied the same analysis to detect stress-dependent fragment accumulation in Rlig1-KO cells by calculating the log_2_FC between stressed Rlig1-KO cells and the mean of all other conditions. In rRNA, we found two distinct cleavage sites with increased NCleavage ratios in the Rlig1-KO stressed condition. The most prominent stress-induced cleavage was detected in the 18S rRNA at position C1767, located in a loop within the 3′ minor domain of 18S rRNA (Fig. 4B). In addition, 5S rRNA showed a cleavage site at C39, situated as well in a loop region (Fig. 4C). For tRNAs, we found stress-specific cleavages in the Rlig1-KO cells in 14 of 39 analysed tRNAs. The cleavages were confined to the anticodon loop, resulting in the accumulation of 5′-PO_4_ end containing 3′tRNA halves (Supplementary Fig. S7C). Stress-induced cleavage sites were further examined with respect to their sequence context within the anticodon loop. We found that 3′ tRNA halves were predominantly generated by cleavage upstream of an adenine residue, with the C-A motif representing the most frequent sequence context (Supplementary Fig. S7D). Furthermore, cleavages occurred mainly at the 3′-site of the anticodon loop (Supplementary Fig. S7E). NCleavage ratios of representative tRNAs cleaved at different positions within the anticodon are shown in Fig. 4D. Interestingly, both tRNA^Gly-CCC2^ and tRNA^Asp-GTC^ again displayed markedly increased NCleavage ratios.

These findings showed that in Rlig1-deficient cells, tRNA and rRNA fragments bearing 5′-PO_4_ termini accumulated under stress conditions. RNA fragments were generated by cleavage at defined structural and sequence contexts, highlighting the role of Rlig1 in maintaining RNA integrity under stress.

## Discussion

RNA repair could serve as a potential alternative to RNA degradation in response to RNA damage. However, beyond the reversal of specific methylations [6] and the resolution of RNA–protein crosslinks [7–9], additional RNA repair pathways in human cells remain elusive. The identification of Rlig1 as the first human RNA ligase capable of catalysing 5′–3′ ligation hinted at the existence of such mechanisms [17]. Although the enzymatic activity of Rlig1 has been characterized previously, its physiological role in defined cellular processes has remained unclear [17, 20–22]. In this study, we investigated the cellular functions of Rlig1 by combining affinity enrichment–mass spectrometry, ribosome association, translation assays and RNA sequencing approaches. Our results identified ribosomes as interaction partners of Rlig1 and suggest a role for Rlig1 in maintaining translational capacity during oxidative stress induced by menadione. Furthermore, 5′-PO_4_ sequencing revealed the accumulation of rRNA and tRNA fragments in Rlig1-deficient cells under menadione-induced stress, supporting a functional link between Rlig1-mediated RNA repair and translational homeostasis.

Affinity enrichment–mass spectrometry using recombinant AMP-Rlig1 or the inactive mutant K57A-Rlig1 as bait identified a diverse set of potential interacting proteins associated with RNA metabolism. Many proteins enriched with the catalytically active AMP-Rlig1 were also detected with the inactive K57A-Rlig1 variant, albeit often at lower levels, suggesting that AMPylation primarily affects interaction strength rather than interactor identity. The overall functional composition of the Rlig1-associated proteome remained largely unchanged under oxidative stress conditions.

Functional annotation of the enriched proteins revealed factors associated with RNA processing, mRNA surveillance, and RNA degradation pathways. Among the enriched proteins were components of the exosome complex, including DIS3, suggesting a possible connection between Rlig1 and RNA processing or degradation pathways. RNA end-processing enzymes such as CLP1 and ANGEL2 were also detected, consistent with the requirement for compatible RNA termini for Rlig1-mediated ligation [44, 45]. Notably, ANGEL2 has previously been shown to cooperate enzymatically with Rlig1 in RNA ligation *in vitro* [17]. While these observations are consistent with a potential role of Rlig1 in RNA processing or repair, it should be noted that these associations were identified using affinity enrichment from lysates and therefore represent potential interactions that require further validation *in vivo*.

Notably, ribosomal proteins were prominently represented amongst the enriched candidates. This observation prompted us to further investigate a potential association of Rlig1 with ribosomal complexes using independent experimental approaches. The association of Rlig1 with ribosomes was confirmed using ribosome sedimentation and *in vitro* crosslinking analyses. These experiments revealed that a fraction of catalytically active Rlig1 associates with ribosomal subunits. Crosslinking suggested preferential interaction with the large ribosomal subunit, although a defined binding interface could not be resolved. Rlig1 did not co-sediment stoichiometrically with ribosomes, indicating that it associates in a partial and likely dynamic likely context-dependent manner.

To put the ribosome–Rlig1 interaction observed *in vitro* into a physiological context, we analysed HEK293 WT and Rlig1-KO cells under oxidative stress conditions. As previously reported, Rlig1-deficient cells were particularly sensitive to menadione treatment, suggesting that the functional role of Rlig1 becomes most apparent under stress conditions [17, 21]. We therefore focused on menadione, a redox-cycling naphthoquinone that generates superoxide radicals through intracellular redox cycling and thereby promotes oxidative stress within the cytoplasm and mitochondria [46–48]. Similar sensitivities of Rlig1-KO cells have been observed for other naphthoquinones, suggesting that Rlig1-dependent effects may be specifically linked to this class of oxidants and their cellular impact [21].

Given the association of Rlig1 with the translational machinery, we examined whether Rlig1 protects translational activity under menadione-induced stress. As expected, menadione treatment resulted in a reduction of global protein synthesis in both WT and Rlig1-KO cell. This is consistent with the effect of oxidative stress on translation reported in the literature [49]. Reactive oxygen species can repress translation through activation of the integrated stress response (ISR) and inhibition of mTOR signalling [49–51]. In addition, oxidative stress can compromise RNA integrity both directly through oxidative nucleobase modifications and indirectly through the activation of stress-responsive ribonucleases that promote RNA cleavage [52–55]. These processes can lead to translational repression and reprogramming of gene expression. Menadione, in particular, has been reported to induce rapid and persistent activation of the ISR even at low concentrations and to stimulate the activity of stress-induced RNases [56, 57]. Notably, menadione-induced translation repression was more severe in Rlig1-deficient cells. Furthermore, polysome profiling revealed enhanced polysome collapse and accumulation of 80S ribosomes, indicative of impaired translation initiation. In addition, we observed that Rlig1 is recruited to the 80S ribosomal fraction under high concentrations of menadione. Together, these findings show that the presence of Rlig1 lowers the extent of stress-induced translational inhibition.

Considering the biochemical activity of Rlig1 as an RNA ligase, we asked whether changes in RNA integrity could be linked to the enhanced translational repression observed in Rlig1-deficient cells. If Rlig1 contributes to RNA repair, its absence would be expected to result in the accumulation of RNA fragments characterized by 5′-PO_4_ termini. In line with this hypothesis, 5′-PO_4_ sequencing revealed the accumulation of defined RNA cleavage products in Rlig1-deficient cells. Cleavage events were confined to specific loop regions within tRNAs and selected rRNA segments. This observation is consistent with previous *in vitro* data showing that Rlig1 preferentially acts on RNAs cleaved within loop regions [17]. The specificity of the observed cleavage sites and sequence motifs suggests the involvement of ribonucleases. While rRNA cleavage products were limited in number, tRNA-derived fragments were abundant and strongly induced under oxidative stress. Supporting the hypothesis that tRNAs represent physiological substrates of Rlig1, tRNA halves have previously been identified as substrates of Rlig1 *in vitro* [22]. Because stress-induced tRNA fragments are known to modulate translation, their preferential accumulation in Rlig1-deficient cells provides a plausible mechanistic link between impaired RNA repair and enhanced translational repression under oxidative stress [55, 58].

Previous work by Hu *et al*. proposed that human cells may possess a tRNA repair mechanism analogous to that described in bacteriophage T4 [22]. Following this model, CLP1 and ANGEL2 could process RNA ends generated by ribonuclease cleavage to produce termini compatible with Rlig1-mediated ligation. Consistent with this, our affinity enrichment analysis identified ANGEL2 and CLP1 as potential interaction partners of Rlig1. In addition, we observed an accumulation of tRNA fragments in Rlig1-KO cells, suggesting that Rlig1 may contribute to the repair of cleaved tRNAs *in vivo*. Taken together, these observations support and extend this model and are consistent with the idea that Rlig1 could function as a transiently ribosome-associated RNA ligase whose activity becomes particularly relevant under oxidative stress conditions. Under such conditions, ribonuclease-induced cleavage events within tRNAs and potentially within specific regions of rRNA may modulate translational activity. Rlig1 could counteract these effects by re-ligating cleaved RNAs, thereby helping to maintain RNA integrity and supporting translational capacity during stress.

While our data provide functional evidence linking Rlig1 to RNA repair and translational control under stress, several mechanistic aspects remain to be clarified. Direct demonstration of ligation at defined endogenous cleavage sites will be necessary to establish the precise substrates of Rlig1 *in vivo*. Furthermore, the nucleases and RNA processing factors that may act upstream or in concert with Rlig1 remain to be identified and confirmed, and the precise causal relationship between rRNA and tRNA fragment accumulation and impaired translation requires further investigation.

Overall, these findings broaden our understanding of RNA repair by identifying Rlig1 as a stress-related RNA ligase involved in translational homeostasis. Further work will be required to elucidate how the interaction between Rlig1 and the ribosome is regulated. Besides ribosomal proteins, several putative interaction partners that are functionally linked to RNA metabolism and repair pathways were detected by affinity enrichment in this study providing an interesting framework for future mechanistic studies.

## Supplementary Material

gkag528_Supplemental_Files

## Data Availability

The lists of significantly enriched proteins of the affinity enrichment coupled to mass spectrometry and the lists of the corresponding enriched GO terms are included in the supplementary information files. Raw data of the affinity enrichment coupled to mass spectrometry, as well as the data bases utilized, and the files of the data analysis are available via ProteomeXchange with the identifier PXD067856. Data of the XL-MS experiment are available via ProteomeXchange with the identifier PXD067615. The raw sequencing data generated in this study have been deposited in the European Nucleotide Archive under project accession number PRJEB110276.
