# Supplementary material for: Human RNA ligase 1 as a novel regulator of ribosome function and translation under oxidative stress
Source: Nucleic Acids Res. 2026 Jun 8;54(11):gkag528. doi: 10.1093/nar/gkag528 (PMC13244148; doi:10.1093/nar/gkag528)
Supplement: gkag528_Supplemental_Files [file gkag528_supplemental_files.zip › Supplementary data.pdf]

MANUSCRIPT TITLE

Human RNA ligase 1 as a novel regulator of ribosome function and translation under oxidative stress

AUTHORS

Florian Michael Stumpf[1,2]†, Marissa Glauner[1,2]†, Jasmin Jansen[2,3], Virginie Marchand[4], Yuri Motorin[4], Florian Stengel[2,3]* and Andreas Marx[1,2]*

[1] Department of Chemistry, University of Konstanz, 78457 Konstanz, Germany

[2] Konstanz Research School Chemical Biology, University of Konstanz, 78457 Konstanz, Germany

[3] Department of Biology, University of Konstanz, 78457 Konstanz, Germany

[4] Université de Lorraine, SMP IBSLor, EpiRNA-Seq core facility, 54000, Nancy

† Florian Michael Stumpf and Marissa Glauner contributed equally to this work

* To whom correspondence should be addressed: E-Mail: andreas.marx@uni-konstanz.de

Correspondence may also be addressed to: E-Mail: florian.stengel@uni-konstanz.de

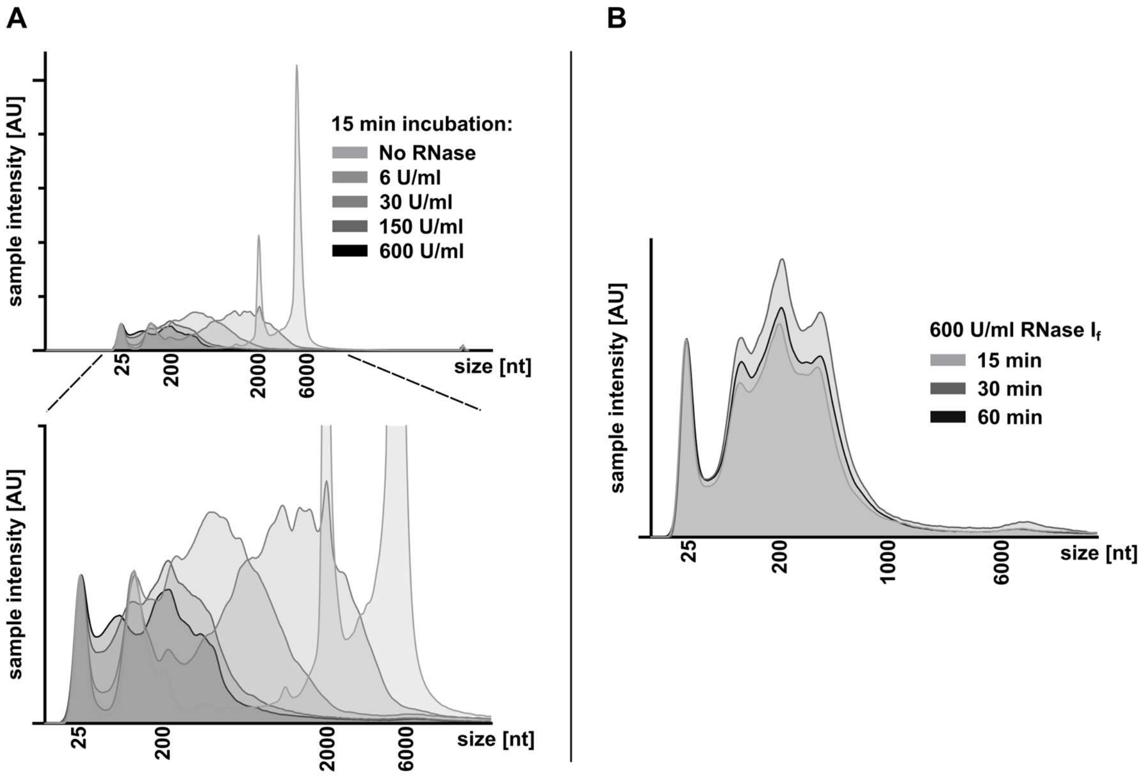

Figure S1: Establishment of the RNA digestion for the affinity enrichment. A) Titration of different concentrations of RNase I$_f$ for optimal digestion with 15 min incubation time. The bottom graph is a close-up of the upper graph. B) Determination of the incubation time needed for an optimal RNA degradation with 600 U/mL RNase I$_f$.

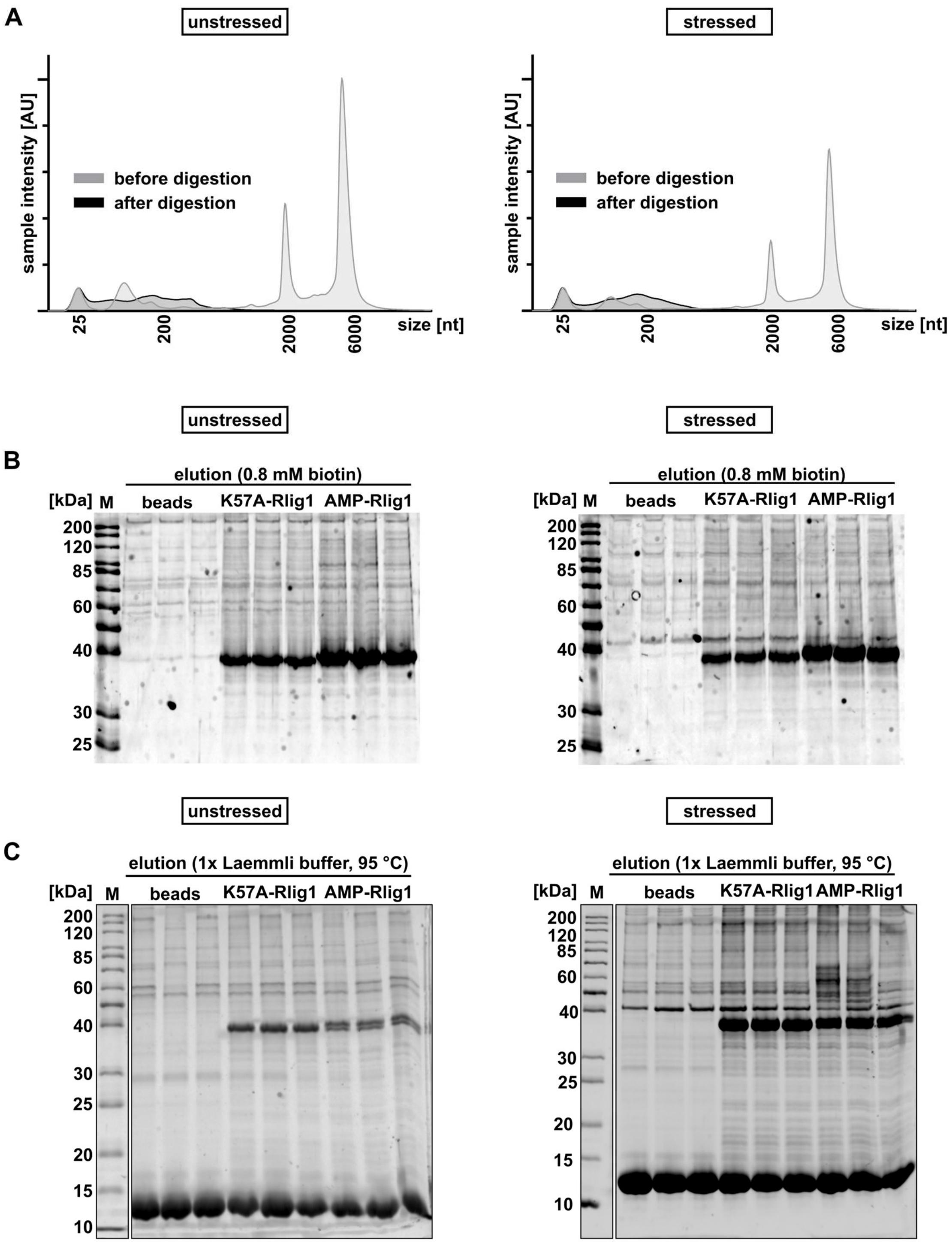

Figure S2: Control of the RNA digestion prior to the affinity enrichment and Krypton-stained gels of the elution samples. A) Exemplary TapeStation 4150 analysis of the RNA in the cell lysates directly after cell lysis (light grey) and of the RNase I_f digested cell lysate directly before application of the lysate to the beads (in black). On the left are the analyses for the unstressed samples, on the right for the stressed samples. B) Krypton-stained gels showing the protein content of the eluted protein samples before trypsinisation. On the left the gel of

the unstressed samples, on the right of the stressed samples. C) Krypton-stained gels showing the protein content that was left on the beads after elution with biotin. The samples here were eluted under denaturing conditions with Laemmli buffer at 95 °C. On the left the gel of the unstressed samples, on the right of the stressed samples.

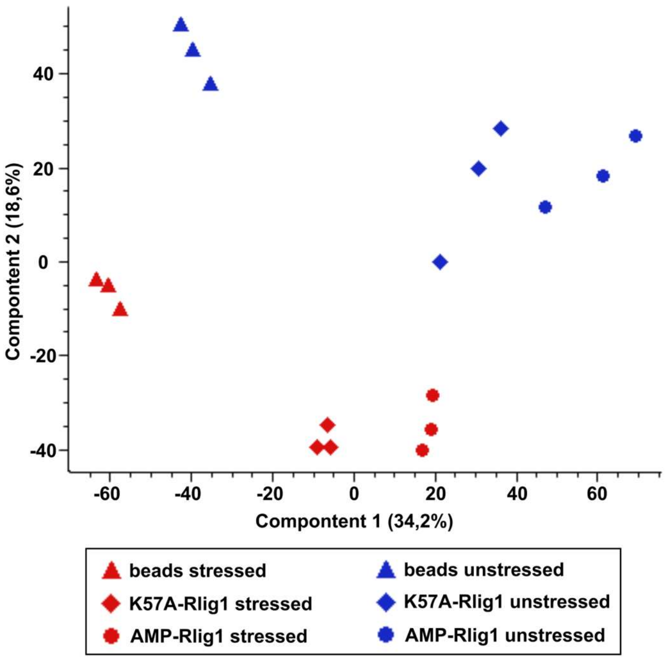

Figure S3: Principal component analysis (PCA) of the identified proteins of the affinity enrichment samples in triplicates. On the X-axis component 1, on the Y-axis component 2. Stressed samples in red, unstressed samples in blue.

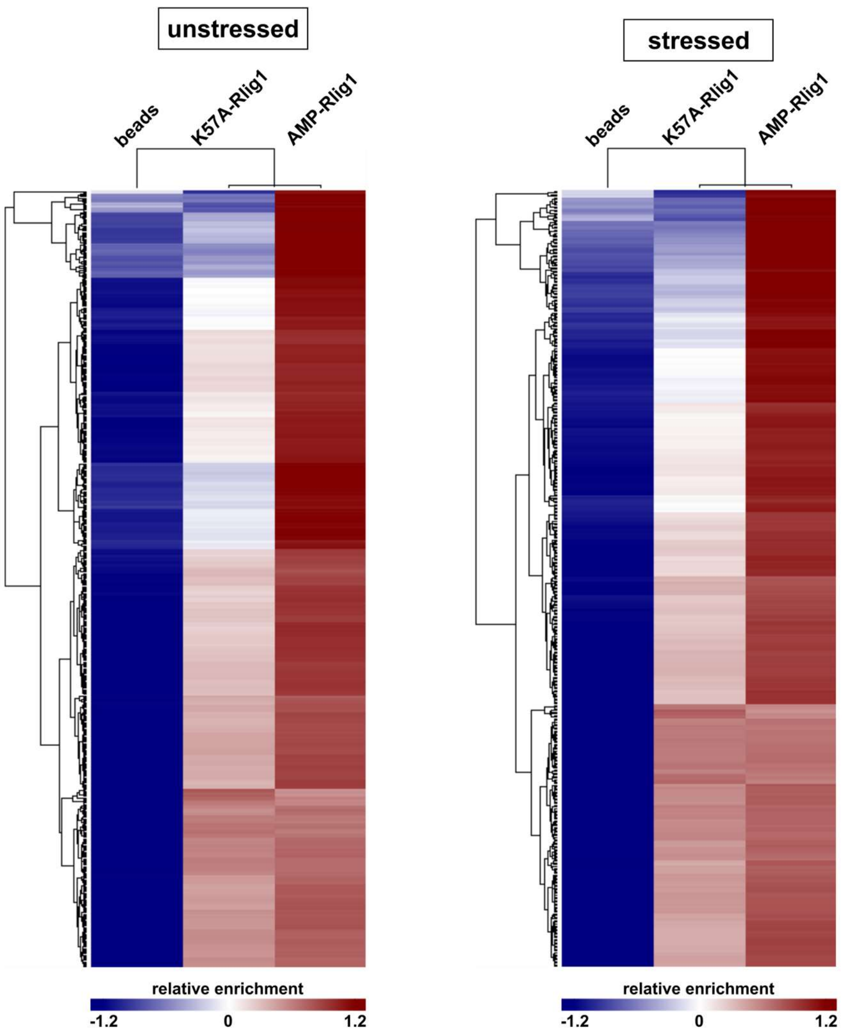

Figure S4: Heatmap of the significantly enriched proteins for AMP-Rlig1. Hierarchical clustering (Euclidean distance) of statistically significantly enriched interactors of AMP-Rlig1 following ANOVA analysis (S0 = 0.1, FDR = 0.01) and Post-Hoc Tukey's test (FDR = 0.01). Only proteins with a minimum Z-score of >0.5 for the AMP-Rlig1 bait and a Z-score <0 for the bead control after the ANOVA analysis are shown. Columns represent the sample type, rows represent an interacting protein. On the left side the heatmap for the unstressed enrichment, on the right side for the stressed enrichment. Colouring signals the relative enrichment of the enriched protein as indicated in the legend.

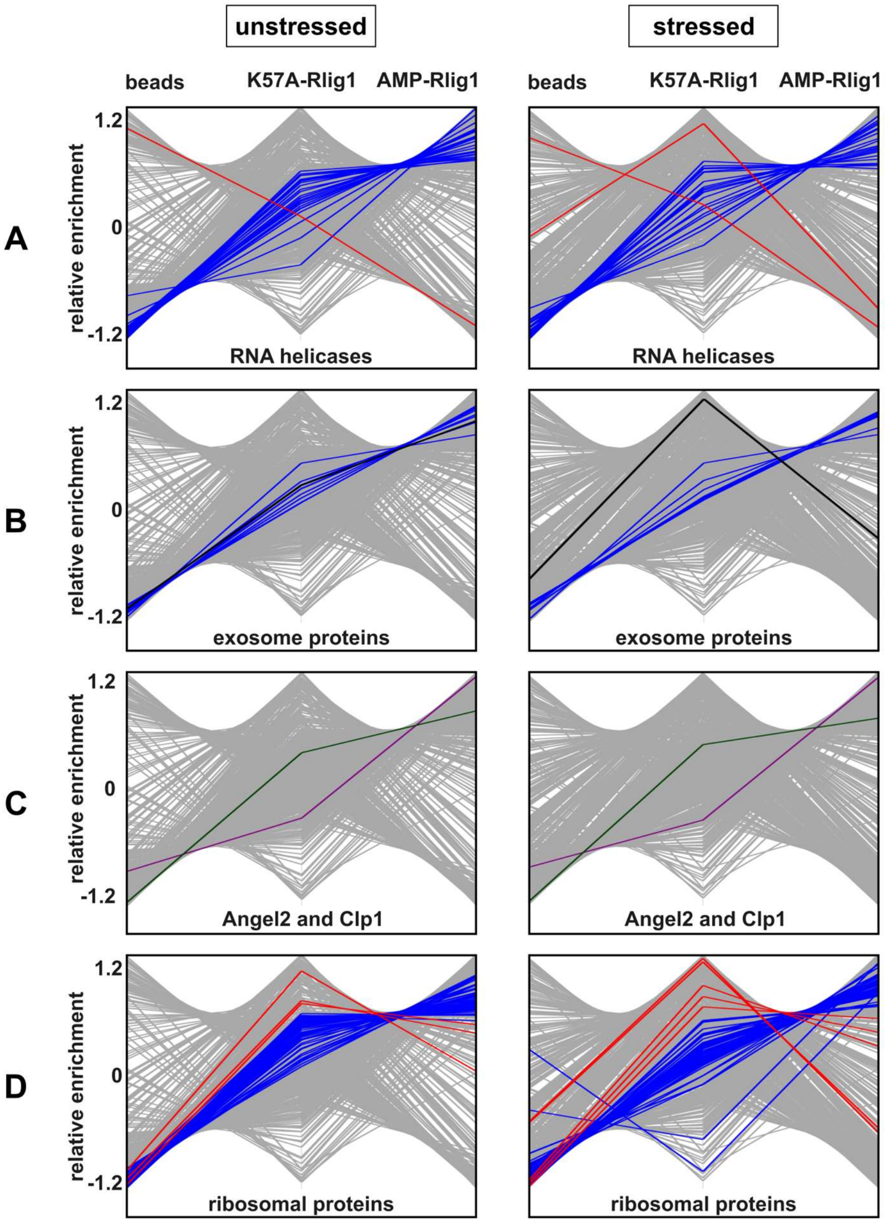

Figure S5: Profile plots of different Rlig1 interacting proteins. Proteins belonging to the group of interest are depicted in colour. Proteins enriched the most for AMP-Rlig1 depicted in blue, proteins enriched the most for K57A-Rlig depicted in red. In the background in grey all the other identified proteins. A) RNA helicases (PG.ProteinDescriptions = RNA helicase). B) Proteins of the exosome. The catalytically active exo- and endoribonuclease DIS3/RRP44 is depicted in black. C) The 2′,3′-cyclic phosphatase Angel2 in green, the RNA kinase CLP1 in violet. D) Cytosolic ribosomal proteins of the large (60S) and the small (40S) ribosomal subunit (PG.ProteinDescriptions = 60S/40S).

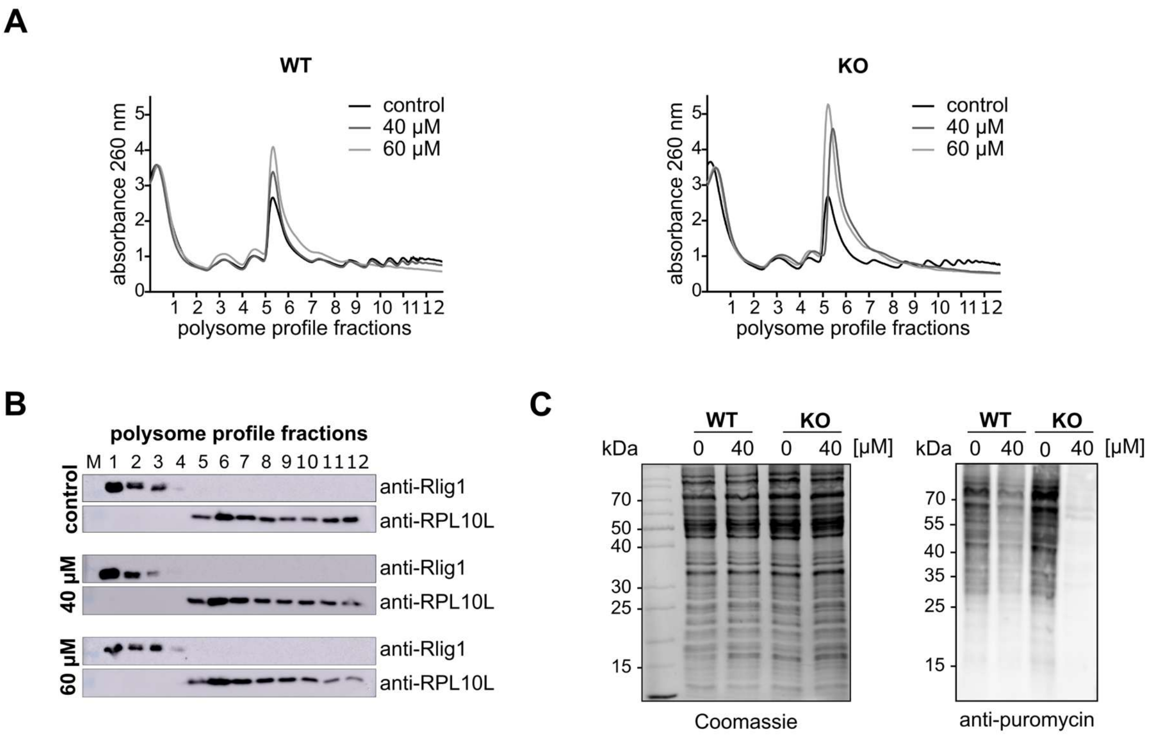

Figure S6: Analysis of polysome profiles and translational activity in HEK293 WT and Rlig1-KO cells under oxidative stress induced by menadione. A) Combined polysome profiles of the WT (left) or Rlig1-KO cells (right) treated with increasing concentrations of menadione. B) Western blot analysis of polysome profile fractions of WT cells treated with different concentrations of menadione. Antibodies against RPL10L as control for the 60S ribosomal subunit and against Rlig1 were used. C) Puromycin incorporation assay in WT and Rlig1-KO cells, either untreated or treated with 40 µM menadione. Total protein levels were visualized by SDS-PAGE followed by Coomassie staining (left), and puromycin incorporation was detected by Western blot using an anti-puromycin antibody (right).

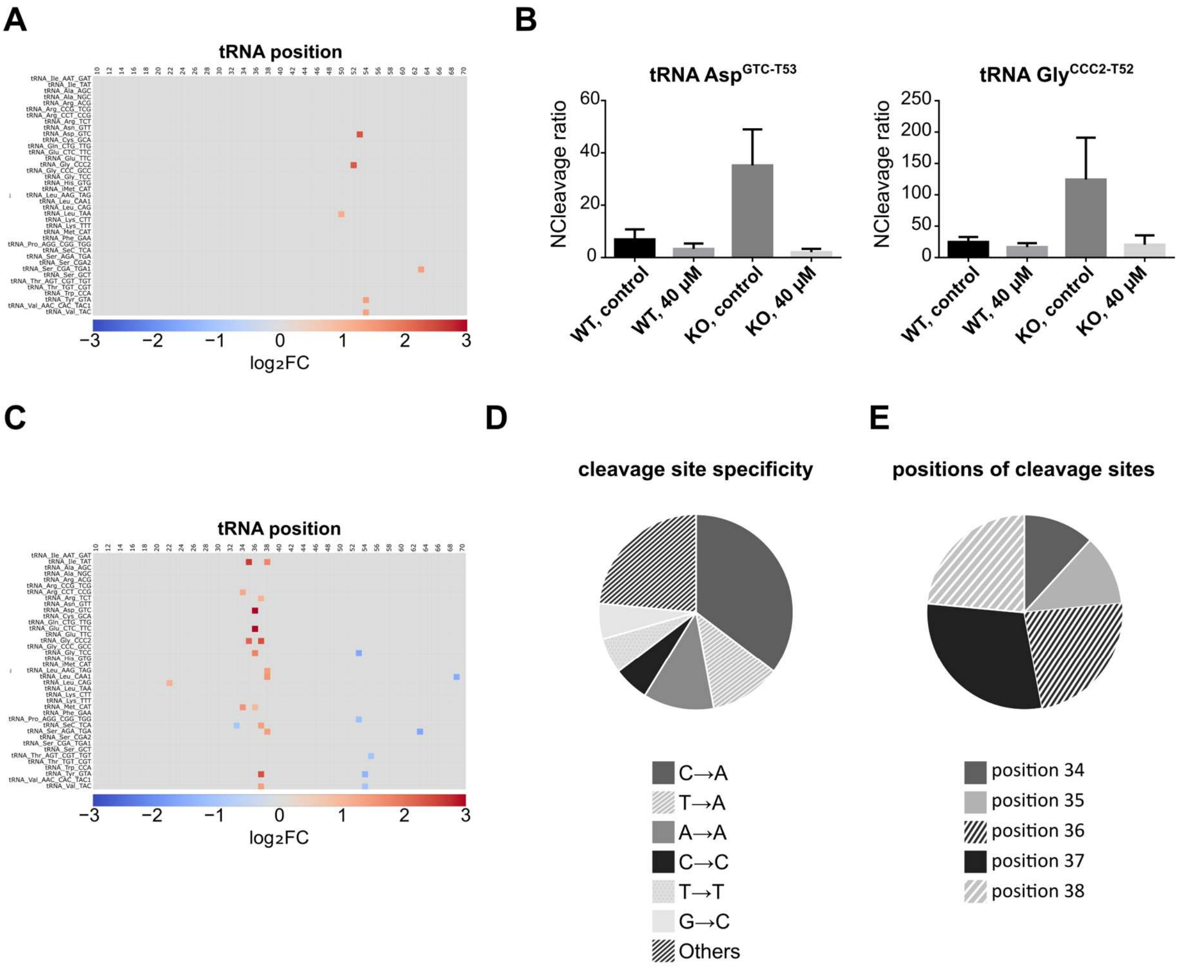

Figure S7: Analysis of accumulated tRNA fragments in Rlig1-KO HEK293 cells. A) Schematic tRNA secondary structure with cleavage sites highlighted (orange stars) (left). Heatmap showing significant $\log_2$FC in NCleavage ratios between WT control and KO control across positions 10–70 nt of all tRNAs ($p < 0.05$, $\log_2$ FC > 1, KO control mean > 25) (right). B) Representative examples of NCleavage ratios of tRNAs with harbouring a cleavage site in the T-loop. NCleavage ratios for tRNA$^{Asp\text{-}GTC}$ at position T53 (right) and tRNA$^{Gly\text{-}CCC2}$ at position T52 (left) are shown for WT and Rlig1-KO cells under physiological conditions (control) or after stress treatment with 40 µM menadione for 180 min (stressed). Data represent five biological replicates. C) Schematic secondary structure of a tRNA with the cleavage site highlighted (orange star, left). Heatmap with filtered $\log_2$FC of NCleavage ratios between KO, 40 µM and control (WT control; WT, 40 µM; KO control) across positions 10–70 nt of detected tRNAs (right). D) Pie chart depicting dinucleotide sequence specificity of significant cleavage events. E) Pie chart showing positional enrichment of significant cleavage events within the anticodon loop.
